# Focal Therapy for Prostate Cancer: State of the Art, Physical Principles, Potentials, and Challenges

**DOI:** 10.3390/cancers18101523

**Published:** 2026-05-09

**Authors:** Luca Orecchia, Simone Steffani, Andrea Micillo, Roberto Miano, Eric Walser, Guglielmo Manenti

**Affiliations:** 1 Department of Surgical Sciences, University of Rome Tor Vergata, 00133 Rome, Italy; 2 Urology Unit, AOU Policlinico Tor Vergata, 00133 Rome, Italy; 3 Diagnostic Imaging Department, AOU Policlinico Tor Vergata, 00133 Rome, Italy; 4 Diagnostic Imaging Unit, Casa di Cura Villa Delle Querce, 00040 Nemi, Italy; 5 Sperling Prostate Center, Delray Beach, FL 33445, USA; 6 Department of Biomedicine and Prevention, University of Rome Tor Vergata, 00133 Rome, Italy

**Keywords:** prostate cancer, focal therapy, multiparametric MRI, index lesion, high-intensity focused ultrasound, irreversible electroporation, cryotherapy, transperineal laser ablation, histotripsy, salvage therapy

## Abstract

Background: The management of localized prostate cancer (PCa) suffers from the dilemma between the overtreatment associated with radical surgery and the uncertainty of active surveillance, highlighting a significant therapeutic gap specifically for intermediate-risk patients and selected low-risk patients. Focal therapy (FT) emerges as an advanced technological solution to balance rigorous oncological control with anatomical and functional preservation. Methods: A narrative review of the literature was conducted to analyze the physical principles underlying various ablative energies (thermal, cryogenic, and non-thermal) as well as radiation-based focal approaches. The review examines the oncological rationale of targeted ablation, recent innovations in imaging, and the expanding clinical scenarios for FT application. Results: Evidence supports the oncological rationale of “Index Lesion” ablation as a targeted curative strategy for clinically significant disease, rather than merely a palliative one. The review highlights the emerging concept of “pushing the disease” and demonstrates the valuable role of salvage focal therapy in the setting of radio-recurrent carcinoma. Furthermore, recent innovations in multiparametric magnetic resonance imaging (mpMRI) and fusion systems have significantly refined patient selection, rendering this minimally invasive approach highly targeted. Conclusions: The current barrier to the universal adoption of focal therapy is the lack of a standardized consensus on the definitions of therapeutic failure and the inadequacy of traditional PSA-based criteria. However, evidence suggests that FT represents a promising, organ-sparing alternative for carefully selected patients with localized PCa, though long-term comparative data are still required.

## Simple Summary

Focal therapy is an emerging treatment for localized prostate cancer that aims to destroy only the tumor-bearing part of the prostate while preserving the surrounding healthy tissue. It was developed to address the gap between active surveillance, which may be insufficient for some patients, and radical treatments, which can cause urinary, sexual, and bowel side effects. This review explains the physical principles behind the main focal therapy techniques, including heat-based, cold-based, electrical, light-activated, and radiation-based approaches, and discusses their current clinical use. The available evidence suggests that focal therapy can provide good cancer control in selected patients while better preserving continence, erectile function, and quality of life. It may also offer a valuable option for men with local recurrence after radiotherapy. However, wider adoption still depends on more standardized definitions of treatment success, failure, and follow-up protocols.

## 1. Introduction

The therapeutic paradigm for localized prostate cancer (PCa) has undergone a profound evolution over the last decade, driven by the necessity to mitigate the limitations of traditional treatments [1,2]. Historically, the management of PCa has relied on a therapeutic dichotomy: on one hand, “whole-gland” therapies, such as radical prostatectomy (RP) and external beam radiation therapy (EBRT), are recognized as the gold standard for tumor eradication but burdened by significant long-term morbidity; on the other hand, active surveillance (AS), is an organ-sparing option reserved for patients with low-risk disease to prevent overtreatment [1,2].

The intrinsic complications and morbidity of radical treatments, particularly urinary incontinence and erectile dysfunction, have catalyzed research towards intermediate solutions [3]. Focal therapy (FT) has evolved from an experimental concept to a potential “middle ground” alternative between active surveillance and radical treatment [1]. The objective of FT is the selective destruction of prostate areas affected by clinically significant neoplasia (csPCa), sparing healthy glandular tissue, the urethral sphincter, the neurovascular bundles, and the rectum [4,5]. This paradigm shift from whole-gland therapy to selective ablation mirrors the evolution already observed in the treatment of other solid malignancies [4,5].

The fundamental catalyst for this transition is represented by advancements in biomedical imaging. The introduction and refinement of multiparametric Magnetic Resonance Imaging (mpMRI) have revolutionized the diagnostic pathway for PCa [5]. Standardized scoring systems, primarily the PI-RADS, have become essential for the millimeter-precise localization of the “Index Lesion”, defined as the primary lesion dictating the patient’s prognosis, and assessment of extracapsular extension, alongside their recently evolved variants, such as PI-RR, for the accurate evaluation of potential local recurrence following previous radical treatments [4,6]. Modern imaging, coupled with image-fusion targeted biopsies (MRI/TRUS fusion), nowadays facilitates the identification of smaller, less aggressive tumors that eluded previous blind systematic biopsy protocols, providing the essential topographical mapping required to plan focal ablation [6,7].

However, the success of FT critically depends on rigorous patient selection [8]. The ideal candidates are men afflicted by organ-confined PCa, generally belonging to the intermediate-risk categories (Gleason Score 3 + 4 or 4 + 3, equivalent to Grade Group 2 and 3) and, in selected cases, low-risk patients with a tumor volume too extensive for AS alone [8,9]. It is imperative that the patient possesses a life expectancy of >10 years, a single disease focus consisting of an index lesion clearly visible on multiparametric imaging, and, most importantly, a strong desire to preserve genitourinary function (potency and continence) while maintaining an optimal quality of life [8,9,10].

Despite the growing body of literature on focal therapy, several aspects of its rationale, technical implementation, and clinical positioning remain incompletely defined. The aim of this narrative review is to summarize the current evidence on focal therapy for localized prostate cancer, focusing on its biophysical basis, available ablative strategies, advances in imaging guidance, criteria for patient selection, and unresolved issues in treatment evaluation. In this context, focal therapy may represent a promising organ-preserving approach for appropriately selected patients.

## 2. Methods

A bibliographic search was performed in March 2026 in the Scopus (Elsevier, Amsterdam, The Netherlands), MEDLINE (US National Library of Medicine, Bethesda, MD, USA), and Web of Science (Thomson Reuters, Toronto, ON, Canada) databases. Combinations of the following descriptors were used according to a free-text protocol: “focal therapy”, “ablative therapies”, “multiparametric magnetic resonance imaging”, “prostate cancer”, “index lesion”, “high-intensity focused ultrasound”, “irreversible electroporation”, “cryotherapy”, and “salvage therapy”, “focal laser ablation”, “histotripsy” and “brachytherapy”. Only articles in English published from the year 2000 were screened for inclusion. Conference abstracts, case reports, and commentaries were excluded. All records were screened by two authors (AM, SS) for inclusion; a third author (LO) was consulted in case of disagreement over inclusion. Results were narratively reported, and no quantitative synthesis of data was performed. Emphasis was placed on studies addressing the rationale for focal therapy, the mechanisms of available ablative modalities, their clinical applications, the possibility of building a personalized treatment framework based on the anatomical location of the lesion, and unresolved issues in post-treatment assessment.

## 3. Biophysics of Ablative Modalities

The clinical implementation of FT utilizes a vast armamentarium of ablative technologies. Although the clinical objective is shared: the irreversible necrosis of the target tumor tissue, the biophysical mechanisms inducing cellular damage differ radically among the various platforms. We can classify these treatments into thermal (high-heat), cold (cryogenic), non-thermal techniques, and radiation-based treatments [11,12,13,14].

### 3.1. Thermal Energies (High-Heat)

#### 3.1.1. High-Intensity Focused Ultrasound (HIFU)

HIFU is the most established focal therapy modality with the longest clinical follow-up [11]. It uses a transrectal transducer to converge high-frequency ultrasound waves (3–4 MHz) onto a precise focal point within the prostate [11,12]. This concentrated energy rapidly raises tissue temperatures to 65–85 °C, causing instant protein denaturation and irreversible coagulative necrosis [11,12]. Additionally, HIFU induces acoustic cavitation: imploding microbubbles mechanically destroy cell membranes and vascular endothelium [13]. Modern systems integrate real-time MR thermometry (MRgFUS) or contrast-enhanced ultrasound (CEUS) to adapt energy dosing in vivo, adjusting for local vascularization and thermal dissipation [12,13,14,15].

#### 3.1.2. Focal Laser Ablation (FLA)

**Figure 1:**
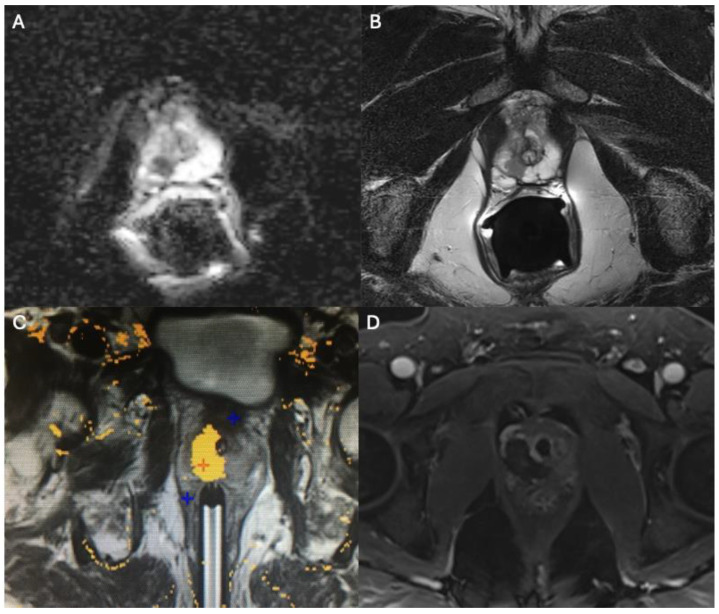
MR-guided focal laser ablation (MR FLA) of a right apical Gleason 3 + 4 prostate cancer lesion. (**A**,**B**) Pre-procedural multiparametric MRI shows the target lesion as hypointense on axial T2-weighted imaging (**A**) with corresponding restricted diffusion on the ADC map (**B**). (**C**) Intraoperative real-time MR thermometry. The yellow overlay indicates the estimated thermal damage zone (>60 °C), demonstrating a highly conformal ablation that completely spares the adjacent urethra. (**D**) Immediate post-ablation axial T1-weighted contrast-enhanced MRI. The non-enhancing region confirms successful ablation of the target lesion and preservation of periurethral tissue.

FLA is an emerging, high-precision thermal therapy that uses a thin, image-guided optical fiber inserted directly into the index lesion [16,17,18]. A near-infrared diode laser (980–1064 nm) emits photons that the tissue absorbs and rapidly converts into heat [14,15]. Unlike HIFU, FLA relies on a highly predictable temperature decay from the fiber tip, yielding precise ablative margins of 1–2 mm [14,19]. Real-time MRI isotherm mapping ensures the protection of critical structures like the urethra and prostatic apex [14,15,19] (Figure 1). Furthermore, the successful use of transperineal laser ablation in benign prostatic disease supports the overall safety, feasibility, and functional tolerability of this approach [20,21,22].

### 3.2. Cryogenic Energies: Cryotherapy

Cryoablation is one of the oldest established treatments for prostate cancer, now refined for focal applications [23]. It utilizes the Joule-Thomson effect (expanding pressurized argon gas through transperineal probes) to rapidly drop tissue temperatures to a lethal −40 °C [23,24]. Cell death is achieved across two rapid-freeze/slow-thaw cycles: osmotic dehydration is followed by intracellular ice crystal formation, which physically destroys the cytoskeleton. Additionally, microvascular thrombosis induces secondary ischemia [23,24,25]. The highly echogenic “ice ball” is monitored in real-time via transrectal ultrasound for spatial control, though the use of urethral warmers and thermocouples remains mandatory to prevent collateral damage to the urethra and rectum [24,25].

### 3.3. Non-Thermal Energies

#### 3.3.1. Irreversible Electroporation (IRE)

**Figure 2:**
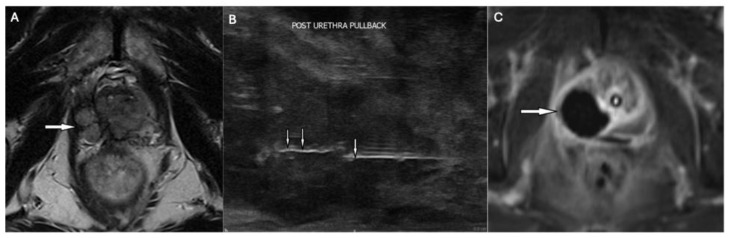
Pulsed electric field (PEF) ablation for recurrent right apical prostate cancer. (**A**) Transverse T2-weighted MRI shows a bilobed recurrence (arrow) one year post-focal laser ablation (Gleason 4 + 3). (**B**) Sagittal transrectal ultrasound during the salvage procedure. Microbubbles highlight the prior PEF needle track (double arrows), with the needle (arrow) retracted for a second activation (50 pulses). (**C**) Post-ablation contrast-enhanced axial T1-weighted MRI demonstrates an avascular ablation zone at the right apex (arrow), confirming accurate targeting.

IRE is an emerging, purely non-thermal technology supported by growing prospective clinical data [26]. The procedure uses 2 to 6 transperineal electrodes to deliver high-voltage (up to 3000 V), microsecond direct current pulses to the target tissue [26,27]. This intense electric field creates permanent nanopores in the cellular phospholipid bilayer [27,28,29]. The resulting loss of ionic homeostasis triggers apoptosis without thermal protein denaturation [27,28,29]. Crucially, IRE targets only cell membranes, sparing the acellular extracellular matrix [27,28,29]. By preserving the vascular and neural scaffolding, IRE offers an excellent safety profile for apical ablations or lesions near the neurovascular bundles, significantly minimizing the risk of erectile dysfunction [26,27,28,29] (Figure 2).

#### 3.3.2. Vascular-Targeted Photodynamic Therapy (VTP)

VTP is a unique systemic-local focal therapy, distinguished as the only modality validated against active surveillance in a phase III trial (PCM301) [30,31,32]. It is indicated for low-risk prostate cancer, forming a distinct clinical niche compared to the intermediate-risk focus of other therapies [29,31,33,34]. The procedure involves intravenously infusing a photosensitizer (Padeliporfin) and subsequently illuminating the target tissue via locally inserted optical fibers (753 nm) [29]. This interaction generates massive quantities of reactive oxygen species (ROS) within the capillaries [29,34]. The primary damage is not directed at the tumor cell, but rather at the endothelial cells of the tumor’s blood vessels, provoking immediate vasoconstriction, platelet activation, and subsequent microvascular thrombosis [34]. Tumoral coagulative necrosis, therefore, occurs secondarily due to induced ischemic necrosis [29,34]. 

#### 3.3.3. Histotripsy

Histotripsy is an emerging noninvasive technique that, while promising, remains currently investigational for prostate cancer treatment, with evidence primarily derived from pre-clinical and ex vivo human specimens [35,36]. Unlike HIFU, it disrupts tissue purely through controlled acoustic cavitation—using specific focused ultrasound parameters to dynamically form and collapse microbubbles—rather than thermal injury [35,36]. By avoiding heat and percutaneous access, this mechanical fractionation theoretically minimizes collateral damage to the urethra, rectum, and neurovascular bundles. While already applied in other organs (liver, kidney) and proven safe in 2018 human trials for benign prostatic hyperplasia (BPH); its application for prostate cancer remains limited to canine models and ex vivo human tissue [35,36,37,38,39]. Therefore, despite a strong theoretical rationale, prospective clinical trials are required to definitively establish its safety, oncologic efficacy, and long-term functional outcomes in human prostate cancer [35,36,40].

### 3.4. Radiation-Based Therapies: Brachytherapy as Focal Therapy

Focal brachytherapy is an emerging application of established radioactive seed or catheter placement, increasingly utilized for targeted gland ablation. Focal brachytherapy utilizes the permanent or temporary insertion of radioactive isotopes to destroy circumscribed tumor areas (Focal or Partial Gland Ablation—PGA) [41,42]. Advanced fusion techniques allow the insertion of seeds (LDR) or catheters (HDR) solely in the affected prostate quadrants or hemi-glands, limiting radiation exposure to the prostatic urethra and the rectal bulb [33]. Recent dosimetry modeling demonstrates the efficacy of this technique in sparing erectile structures and maximizing the dosage to the individual lesion [41,42].

### 3.5. Anatomical-Driven Modality Selection: A Comparative Clinical Framework

While the oncological rationale for focal therapy (FT) is robust, the selection of the specific ablative modality cannot be a “one-size-fits-all” approach [14,29]. Because different energy sources possess unique biophysical properties, urologists must match the specific technology to the topography of the index lesion to optimize safety and efficacy [14,29].

Apical tumors are particularly challenging due to their proximity to the external urethral sphincter and neurovascular bundles [43,44]. In this confined space, non-thermal IRE may be preferred as it preserves the acellular extracellular matrix and neural scaffolding, thereby minimizing the risk of incontinence and erectile dysfunction [45,46,47,48,49,50]. Alternatively, FLA offers a high-precision thermal option; when guided by real-time MR thermometry, it allows for the millimeter-precise margins necessary to spare the adjacent urethra [8,51,52,53].

Treating anterior prostate lesions, especially in larger glands, presents physical challenges. Transrectal HIFU often struggles here because its acoustic waves can lose energy at greater depths or be blocked by calcifications [54,55,56]. Because of this, transperineal methods are generally a much better choice. For example, transperineal cryotherapy easily bypasses depth restrictions to reach the anterior stroma while providing excellent real-time ultrasound visibility [48,50,54,57,58,59].

Conversely, posterior peripheral zone lesions are highly accessible to transrectal HIFU [60,61,62,63]. However, the proximity of the rectal wall necessitates the use of advanced real-time monitoring, such as MRgFUS or CEUS, to prevent fistulas [61,62,64,65]. If cryotherapy is utilized in this region, the use of urethral warmers and thermocouples at the Denonvilliers’ fascia is imperative to mitigate the risk of severe collateral damage [66,67].

Ultimately, effective clinical decision-making in FT relies on recognizing that no single platform is universally superior [12,13,68]. Treatment must be geographically tailored: prioritizing the convergence of maximal oncological effects and preservation of adjacent anatomical structures [13,66,68,69].

## 4. Oncological Rationale and Salvage Setting

### 4.1. The “Index Lesion” Paradigm and the Concept of “Pushing the Disease”

Historically, prostate cancer (PCa) was viewed as a multifocal disease requiring whole-gland treatment, since tumors typically develop synchronously in several areas at once [70,71,72,73,74,75]. However, modern molecular biology and comparative oncology are challenging this traditional approach [76,77]. Just like breast, kidney, or thyroid cancers, which also frequently feature multiple tumor sites but are successfully managed with localized, organ-sparing treatments, we now understand that not all prostate lesions carry the same malignant risk [12,72,78,79,80,81,82] (Table 1).

This shift in understanding centers around the “Index Lesion” theory, which currently is an evolving and debated concept rather than an established rule [83,84,85]. This concept suggests that a patient’s overall prognosis is primarily driven by their largest and most aggressive tumor [50,82,83,86,87]. The oncological premise behind focal therapy is that by targeting and destroying only this primary index lesion, the remaining low-grade “satellite” tumors will likely remain dormant and can simply be safely monitored with standardized imaging [50,73,86]. However, because the long-term biological behavior of these untreated secondary lesions is not entirely predictable and could lead to clinical progression, there is an inherent degree of uncertainty in this approach [83,85,88].

This uncertainty has given rise to the strategic concept of “pushing the disease” [71,89]. Within this framework, focal therapy is not necessarily viewed as a permanent, definitive cure for the entire prostate but rather as a way to safely postpone the need for radical therapies [71,89]. The primary goal is to preserve the patient’s quality of life and minimize toxic side effects during their most active years [11,71,90]. If the untreated lesions eventually progress, the patient has not lost any future therapeutic options, having already benefited from an extended period free of treatment-related comorbidities [11,69,71,90]. While this approach is promising, long-term comparative data to definitively validate the safety and efficacy of “pushing the disease” are still needed [12,13,79,91].

**Table 1:** Occult cancer incidence in the era of focal therapy.

Organ	Study Type	Cohort	Occult-Lesion Rate	Why It Matters for Focal Therapy
Breast	Whole-organ surgical pathology mapping	Holland et al.; 282 invasive T1–2 cancers [92]	Only 37% had no other foci	A single visible breast tumor may underestimate the extent of ipsilateral disease, limiting confidence in purely focal ablation
Breast	Systematic review of autopsy series	Segnan et al.; 8 autopsy case series, 2279 autopsies [93]	Invasive occult cancer: 0–1.5%; DCIS 0.2–14.7%	Supports the concept that the breast harbors a background burden of clinically silent neoplasia
Thyroid	Prospective whole-thyroid mapping after surgery	Park et al.; 82 consecutive patients with solitary PTC on ultrasound [94]	37/82 (45.1%) had additional occult PTC foci; 25/82 (30.5%) had contralateral occult lesions	Directly challenges the assumption that ultrasound-solitary thyroid cancer is truly solitary, especially for focal ablation or lobar approaches
Thyroid	Systematic autopsy whole-gland sectioning	Harach et al.; 101 consecutive autopsies [95]	36/101 (35.6%) had occult papillary carcinoma; 10 glands had 2–5 foci	Shows that multifocal occult thyroid cancer is common even outside clinical disease
Liver	Surgical pathology after resection	Okusaka et al.; 149 patients with solitary HCC ≤ 3 cm [96]	28/149 (19%) had pathologic satellite lesions	Visible HCC may be accompanied by microscopic satellites beyond the imaged edge, supporting wider ablation margins
Liver	Explaining the pathology after transplant	Amara et al.; 919 liver transplants, 790 with known HCC [97]	349/790 (44.1%) had occult multifocal HCC	Confirms that radiology can materially undercount true HCC burden, even within accepted imaging criteria
Lung	Prospective endobronchial staging study	Pierard et al.; 43 patients with 44 visible primary lung cancers [98]	Synchronous early lung cancers in 9.3% of patients	Indicates that selected lung cancer patients may harbor additional occult airway lesions despite visible index tumors

### 4.2. Salvage Focal Therapy in the Setting of Radiorecurrent PCa

One of the emerging and most promising applications of focal therapy is its use as a salvage therapy (salvage focal therapy—SFT) in patients presenting with a local recurrence following primary treatment with radiotherapy (EBRT or brachytherapy) [99,100]. The management of radiorecurrent carcinoma historically represents one of the most complex challenges for the urologist [99,100]. The standard treatment in this scenario is salvage radical prostatectomy (sRP) [99,100]. However, surgical intervention in an irradiated pelvis is associated with high morbidity rates: massive tissue fibrosis obliterates the anatomical cleavage planes, leading to severe urinary incontinence rates approaching 70%, nearly universal erectile dysfunction, and an alarming risk of rectal injury and rectourethral fistula formation (up to 10–15%) [101,102].

In this clinical landscape, SFT offers a valuable alternative [102,103]. By exploiting advanced imaging (mpMRI and PSMA PET/CT) to precisely pinpoint the exact site of intraprostatic recurrence, ablative energies (particularly cryotherapy, HIFU, and IRE) can be utilized to destroy the recurrent focus while sparing the surrounding healthy tissues already compromised by radiation [101,102,103,104]. Recent studies and guidelines (including AUA/ASTRO/SUO directives) have validated SFT, demonstrating that it provides favorable medium-term oncological control (biochemical and clinical progression-free survival) with a drastically lower toxicity profile compared to sRP. SFT minimizes sphincter and rectal damage, suggesting its potential not merely as a palliative treatment but as a feasible second-line option with curative intent [103,105,106,107,108].

## 5. Defining Success and Failure

### 5.1. The PSA Dilemma and the Inadequacy of Traditional Criteria

A primary challenge currently hindering the universal adoption of focal therapy in international guidelines lies in the absence of a standardized and objective consensus for defining therapeutic success and failure [108,109]. Historically, prostate oncology has heavily relied on the kinetics of serum PSA. Following a radical prostatectomy, success is defined by an undetectable PSA (typically defined clinically as maintaining levels <0.1 ng/mL to definitively rule out biochemical recurrence, since consecutive values ≥0.2 ng/mL confirm biochemical recurrence); following radiotherapy, the Phoenix criteria are utilized (PSA Nadir + 2 ng/mL) [110]. In the context of FT, neither of these parameters is biologically applicable or clinically useful [111,112].

Because focal ablation intentionally leaves a significant portion of healthy, secretory prostate tissue intact, the PSA level cannot decrease to zero. The post-FT PSA decline is directly proportional to the volume of the ablated gland and the residual benign epithelial component [50,113]. Consequently, a slow rise in PSA could stem from simple benign prostatic hyperplasia (BPH) in the untreated contralateral lobe, or it could indicate a true oncological recurrence [50,87,114,115]. Establishing an absolute nadir and a standard cut-off limit for post-FT PSA poses a formidable challenge, leading experts to conclude that PSA alone is an unreliable and limited tool for monitoring patients undergoing FT [114,115].

### 5.2. Monitoring with mpMRI and the PI-FAB System

Faced with the limitations of PSA as an isolated marker, modern follow-up relies strictly on the integration of imaging and biopsy [116]. Multiparametric magnetic resonance imaging is recommended at 6–12 months post-treatment to evaluate the ablation zone [116]. To standardize post-treatment reporting, the PI-FAB (Prostate Imaging after Focal Ablation) scoring system has recently been introduced and validated; it classifies post-necrotic and fibrotic alterations to distinguish benign scars from viable residual or recurrent tumor tissue [15]. An area of T2 hypointensity associated with marked restricted diffusion (DWI) and early, asymmetrical post-contrast enhancement (DCE) at the margins of the ablation cavity is a highly suspicious sign of local failure [115,117].

Despite its pivotal role in standardizing post-ablation imaging, the widespread implementation of the PI-FAB scoring system is not devoid of limitations [15,118,119]. Current criticisms within the literature highlight a non-negligible learning curve for uroradiologists and the potential for inter-reader variability, which may affect overall diagnostic reproducibility [118,120,121]. Furthermore, a significant confounding factor resides in the objective difficulty of differentiating early benign post-treatment changes from true residual viable tumor tissue, particularly in early follow-up scans [15,119,120]. Consequently, while the PI-FAB system represents a crucial advancement toward standardization, further prospective validation across larger, multicenter cohorts is required to refine its diagnostic accuracy and mitigate these interpretative challenges [15,118,119].

### 5.3. Defining Recurrence: In-Field vs. Out-of-Field

Another crucial point of debate is the interpretation of histological findings in the event of a post-treatment biopsy (performed due to imaging suspicions or a PSA trigger) [122]. Failure should be categorized topographically and biologically.

- In-Field Recurrence (within the treated field): Indicates a failure to eradicate the clinically significant target tumor (incomplete ablation or insufficient margins) [12].
- Out-of-Field Recurrence (outside the treated field): Indicates the de novo development of new tumor foci or the progression of satellite lesions not previously identified or judged to be indolent at baseline [12].

### 5.4. The Biological Crux

The classification of isolated Grade Group 1 (Gleason 3 + 3 = 6) microfoci detected on a 24-month post-treatment targeted biopsy remains a subject of clinical debate. However, an emerging international consensus, corroborated by extensive multidisciplinary Delphi panels, establishes that the persistence or de novo detection of such indolent lesions does not constitute a therapeutic failure [122,123]. Detecting clinically insignificant PCa does not equate to a failure of FT, since the primary objective was to destroy the biologically aggressive lesion (csPCa, Grade Group 2 or higher) [112]. The mere presence of GG1 does not alter the patient’s life expectancy and falls within the criteria for safe active surveillance [122]. While a universal standard is still being formalized, according to this emerging consensus, true failure is thus solely defined by the discovery of clinically significant PCa (Gleason 3 + 4 or higher) either in-field or out-of-field, which would necessitate the implementation of a repeat focal treatment or a salvage therapy [117].

## 6. Clinical Outcomes and Functional Preservation

The clinical efficacy and safety profiles of various focal therapy modalities have been rigorously evaluated in recent prospective trials and systematic reviews [78,124]. A comprehensive meta-analysis encompassing multiple prospective studies reported a 12- and 24-month clinically significant prostate cancer (csPCa) recurrence-free survival rate of 86% and 81%, respectively [78]. Concurrently, the 5-year radical and systemic treatment-free survival reached 82% [78].

However, while these aggregated outcomes appear highly favorable, they must be interpreted with critical caution. The current literature is characterized by significant heterogeneity in study design, relying predominantly on prospective cohorts with variable follow-up durations rather than long-term randomized controlled trials [12,13,78,80,125]. Furthermore, interpreting and comparing these outcomes across different ablative modalities is inherently complex. This stems from differing patient selection criteria, often blending low- and intermediate-risk disease, and the well-documented lack of a standardized international consensus on what constitutes therapeutic failure or triggers retreatment [13,82,125,126]. Consequently, the reported recurrence-free survival rates reflect a composite of differing institutional follow-up protocols rather than a uniform, standardized clinical endpoint.

Despite these methodological caveats, a key advantage distinguishing focal therapy from radical interventions is the high rate of functional preservation [124]. Post-operative pad-free continence rates consistently range between 92.3% and 100% across all energy modalities [124]. Erectile function preservation is inherently more variable depending on the energy source and the proximity of the index lesion to the neurovascular bundles, with post-treatment potency preservation rates reported up to 94.4% [124]. Furthermore, the overall incidence of severe adverse events remains notably low at approximately 3%. According to the standardized Clavien–Dindo classification, highly adopted in these series, Grade 3 or higher complications (defined as adverse events strictly requiring surgical, endoscopic, or radiological interventions, such as urethral strictures or fistulas) are exceedingly rare, reinforcing the highly favorable safety profile of ablative modalities [12,78].

When analyzing specific technologies, outcomes must be contextualized within their specific cohorts. Modern High-Intensity Focused Ultrasound (HIFU) platforms achieve biopsy negativity rates up to 95% alongside 96–100% continence preservation [127]. Irreversible Electroporation (IRE) demonstrates high tissue-selectivity, achieving 90% in-field ablation of significant cancer while maintaining erectile function in over 85% of cases and demonstrating a 0% rate of de novo incontinence [128]. Furthermore, Focal Laser Ablation (FLA) demonstrates a 15% recurrence rate of csPCa while largely preserving baseline urinary parameters [129]. Ultimately, the true clinical efficacy of these diverse modalities can only be fully appreciated when accounting for the specific study design, patient risk stratification, and definitions of failure applied in each individual cohort [12,13,14,126].

## 7. Economic Sustainability and Cost-Effectiveness

Beyond oncological and functional outcomes, the economic sustainability of these minimally invasive interventions represents a critical metric in modern healthcare resource allocation [130,131]. Recent health-economic evaluations and cost-utility analyses have consistently demonstrated that focal therapy—particularly modalities such as high-intensity focused ultrasound (HIFU) and cryotherapy—exhibits a highly favorable cost-effectiveness profile when compared to standard whole-gland treatments [130]. According to comprehensive Markov models, focal therapy is associated with superior quality-adjusted life year (QALY) gains and a lower overall lifetime cost compared to radical prostatectomy and external beam radiotherapy (EBRT) [130]. This economic advantage is primarily driven by the drastic reduction in treatment-related morbidities; by mitigating the risk of severe genitourinary and gastrointestinal toxicities, focal ablation minimizes the substantial long-term healthcare expenditures required for managing post-operative incontinence, erectile dysfunction, and radiation proctitis [102,130]. Furthermore, emerging cost-utility data on novel image-guided ablative techniques corroborate this trend, supporting focal therapy as a potentially cost-effective strategy within the contemporary paradigm of localized prostate cancer management [131].

## 8. Conclusions and Future Perspectives

Focal therapy for localized prostate cancer continues to mature beyond its initial experimental phase, increasingly demonstrating a favorable safety profile and promising medium-term oncological outcomes. This treatment modality promises to address the ethical and clinical imperative to minimize iatrogenic complications while simultaneously maintaining rigorous oncological control in patients with intermediate-risk disease. The physical principles underpinning the diverse ablative energies currently provide a technological arsenal capable of being tailored to the anatomy of the individual prostate and the specific topography of the index lesion. As evidenced by this review, while short-to-medium-term outcomes are supported by several prospective cohorts, a primary challenge remains the validation of long-term efficacy through randomized controlled trials. Concurrently, there is an urgent need for rigorous methodological standardization of follow-up protocols, radiological scores, and the parameters defining treatment success and failure, transitioning away from the direct application of metrics derived from whole-gland therapies. Urologic oncology is progressively exploring organ-sparing strategies. Treating the dominant tumor lesion rather than the entire organ aligns with the broader goals of precision medicine, provided strict patient selection and rigorous follow-up are maintained. By optimizing patient selection through rigorous protocols and advanced imaging modalities, focal therapy possesses the potential to become a complementary standard of care treatment for a selected cohort of patients, aiding in the transition of localized prostate cancer management toward an even more individualized approach, balancing oncological control with the preservation of functional integrity.

## Data Availability

No new data were created or analyzed in this study. Data sharing is not applicable to this article.

## Abbreviations

The following abbreviations are used in this manuscript:

ADC	Apparent Diffusion Coefficient
AS	Active Surveillance
BPH	Benign Prostatic Hyperplasia
CEUS	Contrast-Enhanced Ultrasound
csPCa	Clinically Significant Prostate Cancer
CT	Computed Tomography
DCE	Dynamic Contrast Enhancement
DCIS	Ductal Carcinoma In Situ
DWI	Diffusion-Weighted Imaging
EBRT	External Beam Radiation Therapy
FLA	Focal Laser Ablation
FT	Focal Therapy
GG	Grade Group
HCC	Hepatocellular Carcinoma
HDR	High-Dose Rate
HIFU	High-Intensity Focused Ultrasound
IRE	Irreversible Electroporation
ISUP	International Society of Urological Pathology
IV	Intravenous
LDR	Low Dose Rate
mpMRI	Multiparametric Magnetic Resonance Imaging
MRgFUS	Magnetic Resonance-guided Focused Ultrasound
PCa	Prostate Cancer
PDT	Photodynamic Therapy
PEF	Pulsed Electric Field
PGA	Partial Gland Ablation
PI-FAB	Prostate Imaging after Focal Ablation
PI-RR	Prostate Imaging for Recurrence Reporting
PSA	Prostate-Specific Antigen
PSMA PET	Prostate-Specific Membrane Antigen Positron Emission Tomography
PTC	Papillary Thyroid Carcinoma
QALY	Quality-Adjusted Life Year
RFS	Recurrence-Free Survival
SFT	Salvage Focal Therapy
sRP	Salvage Radical Prostatectomy
ROS	Reactive Oxygen Species
TRUS	Transrectal Ultrasound
VTP	Vascular-Targeted Photodynamic Therapy
